# *Bifidobacterium* *animalis* subsp. *lactis* BB-12 Primes Epithelial Antiviral Defenses and Restricts Influenza A Virus Replication in Human Intestinal Organoid-Derived Monolayers

**DOI:** 10.3390/microorganisms14040751

**Published:** 2026-03-27

**Authors:** Astghik Stepanyan, Melania Scarpa, Giulia Bernabè, Paola Brun, Anthony Pauletto, Veronica Zatta, Cristiano Salata, Claudia Del Vecchio, Marco Scarpa, Ignazio Castagliuolo

**Affiliations:** 1 Chirurgia Generale 3, Azienda Ospedale-Università Padova, 35128 Padova, Italy; 2 Immunology and Molecular Oncology Diagnostics, Veneto Institute of Oncology IOV-IRCCS, 35128 Padova, Italy; 3 Department of Molecular Medicine (DMM), University of Padova, 35121 Padova, Italy

**Keywords:** probiotics, intestinal organoids, interferon signaling, OAS1, influenza A/H1N1

## Abstract

Viral infections with gastrointestinal involvement remain a significant global health burden with limited therapeutic options. While probiotics show antiviral potential, their impact on primary human intestinal epithelial defenses is poorly defined. This study utilized human intestinal organoid-derived monolayers (ODMs), generated from the non-inflamed mucosa of patients with inflammatory bowel disease, to examine how *Bifidobacterium animalis* ssp. *lactis* BB-12 (BB-12) and *Lacticaseibacillus rhamnosus* GG (LGG) modulate mucosal antiviral pathways. Unlike conventional Caco-2 cells, ODMs preserved physiological cellular diversity and intact innate signaling. Expression of viral receptors and interferon (IFN)-stimulated genes (ISGs) was quantified by RT-qPCR, while the effector 2′-5′-oligoadenylate synthetase 1 (OAS1) was also assessed by immunofluorescence and flow cytometry. Both probiotic strains modulated IFN-associated pathways; however, BB-12 induced a markedly stronger antiviral transcriptional response than LGG. Notably, OAS1 exhibited cell type-specific regulation; while goblet cells showed high basal levels, both probiotics enhanced OAS1 expression selectively in ileal enterocytes. Despite this shared effect, only BB-12 pretreatment significantly restricted Influenza A (H1N1) replication in ileal ODMs, whereas LGG did not significantly affect viral replication. These findings establish human ODMs as a superior platform for probiotic immunology, suggesting that BB-12 more effectively shapes epithelial antiviral “set-points” and highlighting OAS1 as a sensitive component of a broader antiviral program.

## 1. Introduction

Viral infections of the gastrointestinal tract represent a major cause of morbidity worldwide, particularly in vulnerable populations [1,2]. Beyond classical enteric pathogens, the gut is increasingly recognized as a site of replication for viruses typically associated with other organ systems, such as Influenza A virus (IAV) [3,4] and SARS-CoV-2 [5]. Clinical evidence of gastrointestinal symptoms in IAV patients, coupled with the detection of viral RNA in stool, suggests that the human gut serves as an underappreciated reservoir and site of replication [6,7], a phenomenon often described within the “gut–lung axis” framework [8].

Probiotics—live microorganisms that confer health benefits [9]—have emerged as promising modulators of mucosal immunity, with in vivo evidence of antiviral activity [10,11,12]. The proposed mechanisms driving these antiviral effects are multifaceted. They encompass direct viral antagonism through competitive exclusion for epithelial adhesion sites, the secretion of virucidal metabolites (such as bacteriocins and organic acids), and complex cross-talk with the host immune system, including the enhancement of secretory IgA and the activation of natural killer (NK) and T-cells [13,14]. However, while systemic immune modulation and direct microbe-virus interactions are well-documented, the specific epithelial-intrinsic antiviral pathways primed by probiotics remain poorly defined. Among the numerous probiotic candidates, *Bifidobacterium animalis* ssp. *lactis* BB-12 (BB-12) and *Lacticaseibacillus rhamnosus* GG (LGG) were selected as benchmark strains due to their extensive clinical documentation, safety profile, and reported immunomodulatory properties, making them suitable reference probiotics for mechanistic epithelial studies [15]. Their inclusion also enabled a comparison between representative strains from the *Bifidobacterium* and *Lacticaseibacillus* genera with known gastrointestinal relevance. However, most studies have relied on immortalized cell lines like Caco-2, which lack the cellular complexity (e.g., goblet cells, Paneth cells) and the robust innate immune signaling found in primary tissue [16,17].

To overcome these limitations, human intestinal organoid-derived monolayers (ODMs) have emerged as advanced experimental models that preserve epithelial cellular diversity and maintain intact type I/III interferon (IFN) signaling [18,19,20,21,22,23]. These systems therefore provide a physiologically relevant platform to investigate epithelial–microbe–virus interactions in the human intestinal mucosa.

In this study, we hypothesized that transient exposure to benchmark probiotics (BB-12 and LGG) directly primes the innate antiviral state of the human intestinal epithelium in a strain- and cell type-specific manner, thereby restricting subsequent viral replication. By utilizing human ODMs to model a vulnerable epithelial barrier, we aimed to dissect the distinct transcriptional programs induced by these strains and to identify specific epithelial biomarkers of probiotic-mediated antiviral defense against Influenza A (H1N1).

## 2. Materials and Methods

### 2.1. Intestinal Crypt Isolation and Organoid Culture

Collection and culture of human ileal and colonic tissues from patients with inflammatory bowel disease (IBD) received institutional review board approval (Ethics Committee of the Azienda Ospedaliera of Padova, Italy, project title: “Prospective study of predictors of fibrotic recurrence in Crohn’s disease”; Project No. 3570bis/AO/15) and the study was performed according to the principles of the Declaration of Helsinki. Clinical characteristics of the biological donors used in this study, including age, sex, and disease status, are provided in Supplementary Table S1. Crypt isolation was performed as previously described, using a combination of two protocols [24,25]. Briefly, dissected mucosa was trimmed using a sterile scalpel and washed in Hanks’ Balanced Salt Solution (HBSS; Gibco™, Thermo Fisher Scientific, Waltham, MA, USA) using a 10 mL serological pipette precoated with 10% (vol/vol) fetal bovine serum (FBS; Gibco™). Tissue fragments were incubated in 2.5 mM EDTA/HBSS for 40 min at 4 °C with gentle rocking. Crypts were released by vigorous pipetting, pooled from fractions with the highest crypt/villi ratio, centrifuged at 1200 RPM for 5 min at 4 °C and resuspended in ice-cold Matrigel (Corning Inc., Corning, NY, USA) at 20 µL per 100 crypts. Domes (20 µL) were seeded in pre-warmed 48-well plates, polymerized at 37 °C for 10 min, and overlaid with IntestiCult™ Organoid Growth Medium (OGM; STEMCELL Technologies, Vancouver, BC, Canada) supplemented with 1% antibiotic-antimycotic solution (Gibco™) and 10 µM Rho kinase inhibitor (RhoKi, Y-27632, STEMCELL Technologies) restricted to the first 48 h post-seeding. Cultures were maintained at 37 °C, 5% CO2, with medium replaced every 2–3 days. Mature organoids were observed within 7–10 days.

### 2.2. Organoid Passaging and Monolayer Differentiation

Organoids were released from Matrigel using Trypsin-EDTA solution (Biowest, Nuaillé, France) and mechanically dissociated. Fragments were collected in ice-cold DMEM—F12 (Biowest) supplemented with 10% FBS, centrifuged and reseeded in fresh Matrigel. For monolayer differentiation, organoids were dissociated into single cells using Gentle Cell Dissociation Reagent (STEMCELL Technologies) for 5 min at room temperature, washed, centrifuged, and resuspended in IntestiCult™ Organoid Differentiation Medium (ODM; STEMCELL Technologies) with 1% AA and 10 µM RhoKi restricted to the first 48 h post-seeding. Cells were seeded in 96-well plates precoated with DMEM/F12 containing 5% Matrigel and allowed to differentiate for 48 h before medium replacement. To ensure the transition from 3D organoids to a functional 2D barrier, monolayer confluence was monitored via phase-contrast microscopy. Differentiation was confirmed after 96–120 h in ODM by the presence of mature cell markers (SI, MUC2) via immunofluorescence. Monolayer viability following probiotic exposure was assessed by monitoring medium pH and epithelial cell morphology; no significant cytopathic effects or acidification-induced detachment were observed at the used MOI and time points. Differentiation timing and marker expression were consistent across independent donors and passages used in this study.

### 2.3. Probiotic Strains and Culture Conditions

*Lactobacillus rhamnosus* GG (LGG; ATCC BAA-3227) and *Bifidobacterium animalis* subsp. *lactis* BB-12^®^ (BB-12; ATCC BAA-2848) were obtained from the American Type Culture Collection (ATCC, Manassas, VA, USA). Strains were cultured in Man–Rugosa–Sharpe (MRS) broth as previously described and stored in PBS-glycerol 20% at −80 °C until use [17]. Frozen stocks were cultured in MRS broth under anaerobic conditions at 37 °C for 18 h. Bacterial growth was monitored via OD_600_. Cultures were centrifuged (5000 RPM, 5 min), washed in PBS, and resuspended at 10^8^ CFU/mL for experiments.

### 2.4. Organoid-Derived Monolayer Treatments

Differentiated monolayers (>80% confluence) were washed twice with D-PBS and exposed to probiotics at a multiplicity of infection (MOI) of 10 for 3 h in antibiotic-free ODM. This concentration was chosen to simulate transient mucosal exposure while maintaining epithelial viability and medium pH stability. Monolayers were subsequently washed twice with D-PBS to remove non-adherent bacteria, and further maintained in ODM supplemented with 1% antibiotic-antimycotic (AA) prior to downstream analysis or viral challenge.

### 2.5. RNA Extraction and Quantitative Real-Time PCR

Total RNA was isolated using the E.Z.N.A. Total RNA Kit I (Omega Bio-Tek Inc., Norcross, GA, USA) with on-column DNase digestion. For monolayer gene expression analysis, cDNA synthesis and amplification were performed with the iTaq™ Universal SYBR^®^ Green One-Step RT-qPCR Kit (Bio-Rad Laboratories, Inc., Hercules, CA, USA) on a QuantStudio™ 3 instrument (Applied Biosystems, Thermo Fisher Scientific, Carlsbad, CA, USA). Cycling conditions were: 50 °C for 10 min, 95 °C for 1 min, followed by 40 cycles of 95 °C for 15 s, annealing/extension temperature °C for 60 s. Primer sequences, annealing/extension temperatures and concentrations are listed in Table 1. Gene expression levels were normalized to the *ACTB* housekeeping gene. For viral replication assessment, quantitative RT-PCR was performed using the AgPath-ID™ One-Step RT-PCR Kit (Applied Biosystems) on the same instrument. Viral copy number was determined from a standard curve generated with serial dilutions (10^1^–10^6^ copies/reaction) of a plasmid containing the influenza A virus (IAV) amplicon. The following primers and probe were used: forward 5′-GACCRATCCTGTCACCTCTGAC-3′, reverse 5′-AGGGCATTYTGGACAAAKCGTCTA-3′, probe 5′-FAM-TGCAGTCCTCGCTCACTGGG-CACG-BHQ1-3′. Cycling conditions were: 50 °C for 10 min, 95 °C for 10 min, followed by 40 cycles of 95 °C for 15 s, 60 °C for 60 s.

### 2.6. Immunofluorescence Microscopy

Organoid-derived monolayers were differentiated in 8-well cell culture chambers. Cells were fixed in 4% paraformaldehyde (Sigma-Aldrich, St. Louis, MO, USA) for 10 min at room temperature, permeabilized with 0.1% Tween-20 PBS ( Thermo Fisher Scientific, Waltham, MA, USA) for 5 min, and blocked in 0.1% Tween-20, 2.5% bovine serum albumin (BSA) PBS for 1 h. Primary antibodies (Table 2) were applied overnight at 4 °C or 1 h at room temperature, followed by fluorophore-conjugated secondary antibodies (Table 2) for 1h at RT. Nuclei were stained with Draq5 (1:10,000). Chambers frames were removed and slides were mounted with FluorSave™ (Merck Millipore, Burlington, MA, USA). Images were acquired with Nikon Eclipse Ti Confocal Microscope (Nikon Instruments Inc., Tokyo, Japan) and analyzed using ImageJ software v1.54p (National Institutes of Health, Bethesda, MD, USA).

### 2.7. Flow Cytometry

Monolayer-derived cells were washed with D-PBS and detached using Gentle Cell Dissociation Reagent (STEMCELL Technologies, 2000 rpm for 5 min at 4 °C). Pellets were resuspended in Fixation Buffer (eBioscience, Thermo Fisher Scientific, Waltham, MA, USA) and incubated for 30 min at room temperature (RT). Cells were then permeabilized with Permeabilization Buffer (Invitrogen) for 20 min at RT. Following permeabilization, cells were incubated overnight at 4 °C with PE-conjugated anti-OAS1 antibody together with either Alexa Fluor 488-conjugated anti-MUC2 antibody or rabbit anti-sucrase-isomaltase (SI) antibody in Permeabilization Buffer (eBioscience). After washing, cells stained with rabbit anti-SI were incubated with Alexa Fluor 647-conjugated anti-rabbit secondary antibody for 1 h at RT. Antibody details and working concentrations are provided in Table 2. Following final washes, cells were resuspended in PBS and analyzed on a BD^®^ LSR II SORP flow cytometer (BD Biosciences, Franklin Lakes, NJ, USA). Data was processed using FlowJo™ v10 Software (BD Biosciences, Ashland, OR, USA).

### 2.8. Influenza A/H1N1 Infection and Titration

Influenza A (H1N1) virus was obtained from ATCC (VR-1469™). The virus was propagated in MDCK cells (CCL-34, ATCC) grown in DMEM supplemented with 0.3% BSA (Sigma-Aldrich) and 1 µg/mL (TPCK)-trypsin (Sigma-Aldrich). Infected MDCK cells were incubated at 37 °C and monitored daily for cytopathic effect (CPE). Viral titers were determined by fluorescent focus assay [26] using FITC-conjugated anti-NP antibody (Argene, bioMérieux, Marcy-l’Étoile, France). Viral stocks were stored at −80 °C until use.

ODMs were infected with Influenza A/H1N1 at MOI 1. After 2 h, viral inoculum was removed and ODM was added. Cell culture supernatants were collected for viral RNA quantification using the AgPath-ID™ One-Step RT-PCR kit. Concurrently, monolayers were fixed for immunofluorescence analysis of the viral nucleoprotein (NP). Images were acquired using a Nikon Eclipse Ti microscope with a 60× oil-immersion objective. For each experimental condition, 5–8 randomly selected, non-overlapping fields were captured. The percentage of NP-positive cells was quantified using ImageJ/Fiji software v1.54p by normalizing the number of NP-stained cells (identified by fluorescence intensity above a set threshold) to the total number of Hoechst-stained nuclei.

To evaluate the prophylactic effect of probiotics, ODMs were pre-exposed to BB-12 or LGG (MOI 10) for 3 h in antibiotic-free medium. Following treatment, probiotics were removed and monolayers were washed twice with D-PBS to eliminate non-adherent bacteria. Monolayers were then challenged with Influenza A/H1N1 (MOI 1) for 2 h at 37 °C. After the viral adsorption period, the inoculum was replaced with fresh differentiation medium, and cultures were maintained for 24 h before proceeding with viral RNA quantification in the supernatant and NP-staining analysis, as described above.

### 2.9. Statistical Analysis

Experiments were performed in at least two technical replicates per condition with three independent biological replicates. Statistical significance was determined using either Student’s *t*-test or ANOVA with post hoc comparisons. Gene expression is shown as fold change relative to untreated controls. Data are presented as mean ± standard error of the mean (SEM). A *p*-value < 0.05 was considered statistically significant. All statistical analyses were performed using GraphPad Prism 9.5.0 (GraphPad Software, San Diego, CA, USA).

## 3. Results

### 3.1. Human Intestinal Organoid-Derived Monolayers Exhibit Physiological Epithelial Diversity

**Figure 1:**
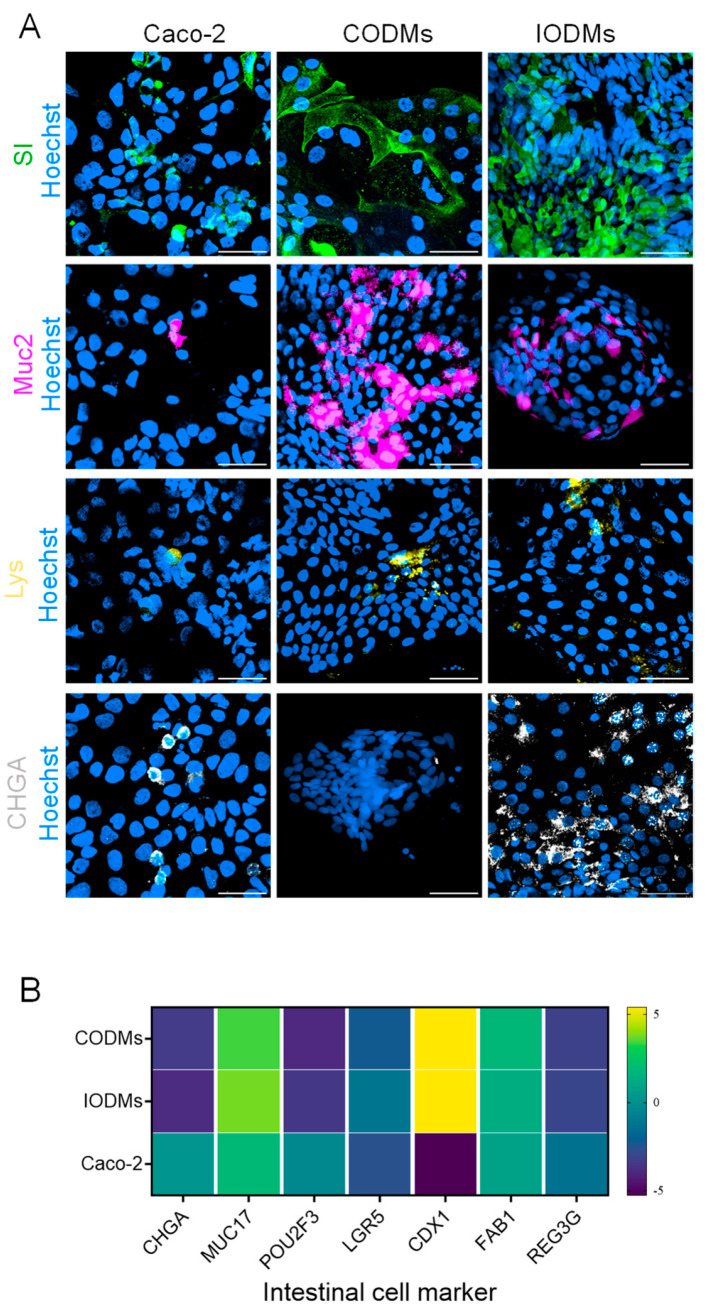
**Characterization of human intestinal organoid-derived epithelial monolayers.** (**A**) Representative immunofluorescence images of differentiated colonic (CODMs) and ileal (IODMs) monolayers compared to the Caco-2 cell line. Lineage markers: Sucrase-Isomaltase (SI, enterocytes, green), Mucin 2 (MUC2, goblet cells, pink), Lysozyme (LYS, Paneth cells, yellow), and Chromogranin A (CHGA, enteroendocrine cells, gray). Nuclei: Hoechst (blue). Scale bars, 50 μm. (**B**) Heatmap showing the relative expression of intestinal epithelial lineage-specific genes measured by RT-qPCR in Caco-2, CODMs, and IODMs. Data are presented as log_10_(2^−ΔCt^) values. Lineage markers: *CHGA* (enteroendocrine cells), *MUC17* (goblet cells), *POU2F3* (Tuft cells), *LGR5* (stem cells), *CDX1* (enterocytes), *FABP1* (early enterocytes) and *REG3G* (Paneth cells). Results represent three independent biological donors (*n* = 3) with at least two technical replicates.

To establish a physiologically relevant system, we generated organoid-derived monolayers (ODMs) from human ileal (IODMs) and colonic (CODMs) tissues. While Caco-2 consists of a relatively homogeneous population of enterocyte-like cells, ODMs successfully recapitulated the multi-lineage composition of the human intestinal epithelium (Figure 1). Immunofluorescence confirmed the presence of Sucrase-Isomaltase (SI)-positive enterocytes, Mucin 2 (MUC2)-positive goblet cells, and Lysozyme (LYS)-positive Paneth cells (Figure 1A). Notably, expression of the stem-cell marker *LGR5* is low in ODMs, consistent with the expected reduction in stem-cell signatures following epithelial differentiation into monolayers (Figure 1B). While CODMs displayed a higher proportion of Paneth cell markers—likely reflecting the stable mucosal remodeling characteristic of the IBD-derived background [27]—the overall transcriptomic profile confirms that ODMs maintain differentiated and heterogeneous epithelial composition, making them a more physiologically relevant model for studying lineage-specific host–microbe interactions than standard immortalized cell lines (Figure 1B).

### 3.2. Probiotic Strains Elicit Distinct Antiviral Transcriptional Responses

**Figure 2:**
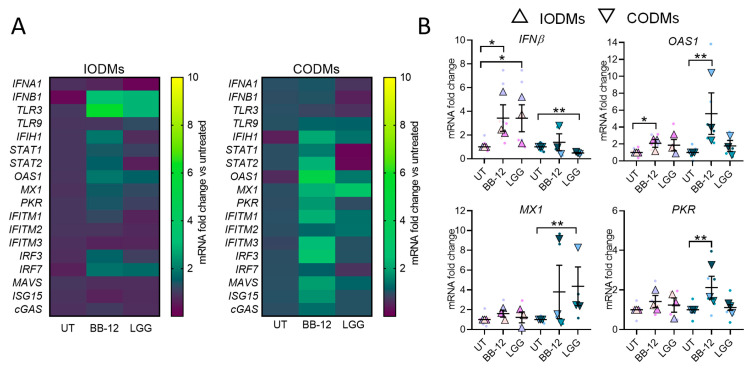
**Probiotic-driven induction of the epithelial antiviral program.** (**A**) Heatmap showing transcriptional changes in ODMs following exposure to BB-12 or LGG (MOI 10). Genes analyzed include viral recognition receptors (*IFIH1*, *TLR3*, *TLR9*), signaling mediators (*IRF3*, *IRF7*, *MAVS*, *STAT1*, *STAT2*), type I interferons (*IFNA1*, *IFNB1*), and interferon-stimulated genes (*EIF2AK2/PKR*, *IFITM1–3*, *OAS1*, *MX1*). (**B**) Quantitative RT-qPCR of selected key effectors antiviral genes (*OAS1*, *MX1*, *IFNB1*, *EIF2AK2/PKR*). Data are expressed as fold change relative to untreated controls (2^−ΔΔCt^). Small circles represent individual technical replicates and large triangles represent the mean of each independent biological donor (*n* = 3). Bars represent mean ± SEM. Statistical significance was determined by two-way ANOVA with Tukey’s post hoc test; * *p* < 0.05, ** *p* < 0.01.

We next evaluated the capacity of BB-12 and LGG to modulate antiviral pathways. Both strains induced a broad set of genes involved in viral recognition and IFN signaling, with BB-12 eliciting a more robust response (Figure 2A). In IODMs, BB-12 treatment significantly upregulated *IFNB1* and several downstream interferon-stimulated genes (ISGs), including *OAS1* and *MX1* (Figure 2B). The observation that certain ISGs remained elevated even when IFN transcripts showed modest induction likely reflects the distinct temporal kinetics of epithelial innate immune signaling, where a transient interferon induction can lead to sustained expression of downstream interferon-stimulated genes due to amplification within the antiviral signaling cascade.

### 3.3. Cell Type-Specific Modulation of OAS1 Expression

**Figure 3:**
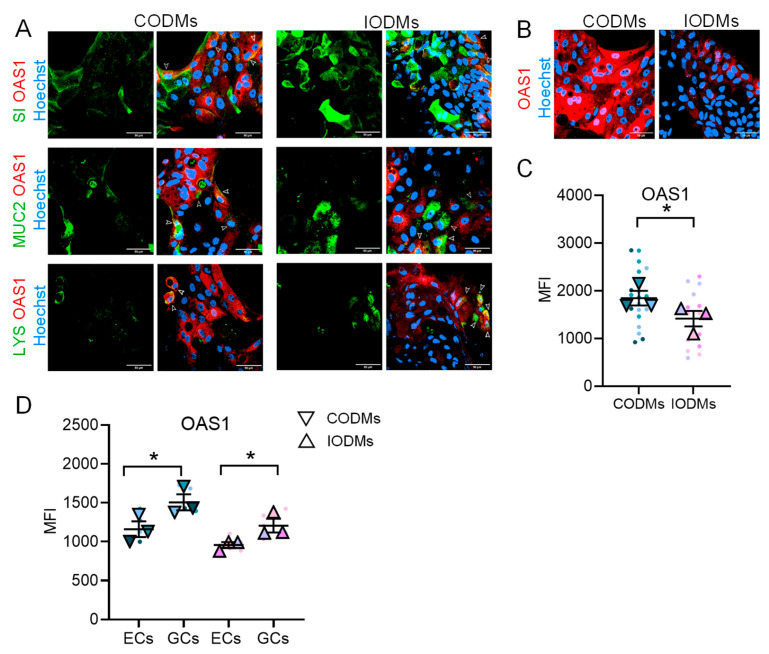
**Basal cell type-specific distribution of OAS1.** (**A**) Co-localization of OAS1 (red) with SI, MUC2, or LYS (green) in ODMs. Nuclei were counterstained with Hoechst (blue). Scale bars, 50 μm. (**B**) Representative images illustrating basal OAS1 expression levels in CODMs and IODMs. (**C**) Quantification of OAS1 median fluorescence intensity (MFI) from immunofluorescence images analyzed with ImageJ. (**D**) Flow cytometry analysis of basal OAS1 expression in SI^+^ enterocytes (ECs) and MUC2^+^ goblet cells (GCs). Quantitative data in (**C**,**D**) are shown as superplots (small circles represent individual technical replicates and large triangles represent the mean of each independent biological donor, *n* = 3). Bars represent mean ± SEM. Data represent background-subtracted MFI. Statistical analysis was performed by two-way ANOVA with Tukey’s multiple comparisons test (* *p* < 0.05).

**Figure 4:**
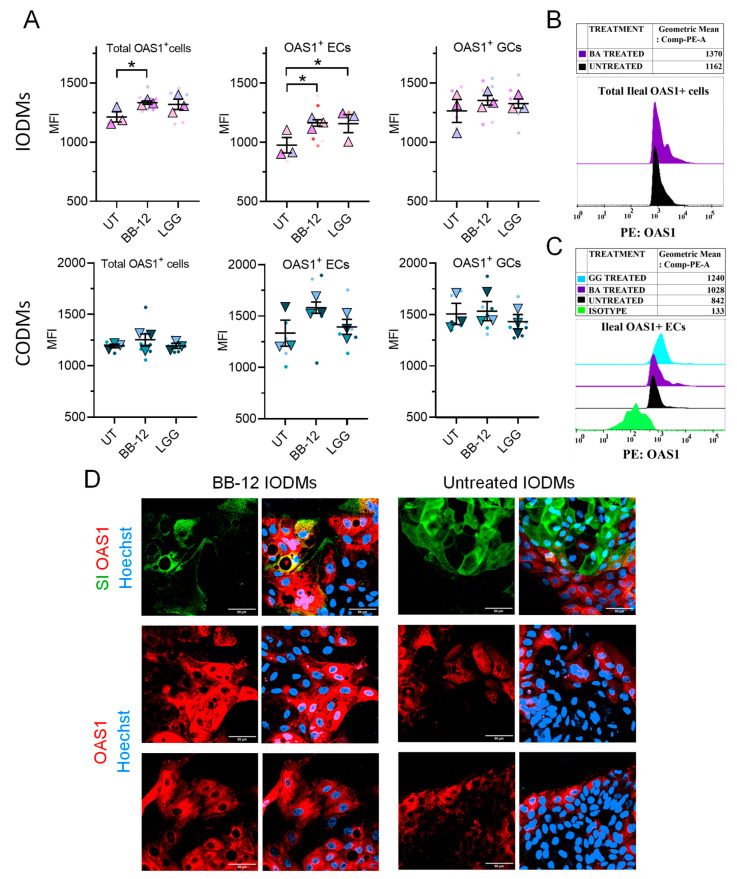
**Targeted modulation of OAS1 by BB-12.** (**A**) Flow cytometry quantification of OAS1 mean fluorescence intensity (MFI) in SI^+^ enterocytes and MUC2^+^ goblet cells from colonic (CODM) and ileal (IODM) monolayers. MFI values were background-subtracted using isotype controls. (**B**) Flow cytometry histograms showing the percentage of total OAS1^+^ cells in untreated versus BB-12-pretreated ileal monolayers. (**C**) Flow cytometry analysis of OAS1^+^ enterocytes in untreated, BB-12-, and LGG-treated ileal monolayers. (**D**) Representative confocal images of ileal monolayers showing OAS1 (red) and SI (green) after BB-12 treatment. Nuclei were counterstained with Hoechst (blue). Scale bars, 50 μm. Quantitative data are presented as superplots (small circles: technical replicates; large triangles: biological donor means, *n* = 3). Bars represent mean ± SEM. Statistical significance was determined by one-way ANOVA with Tukey’s post hoc test; * *p* < 0.05.

The use of primary ODMs allowed us to dissect the cellular distribution of OAS1, an effector that would appear uniformly expressed in a Caco-2 monolayer. OAS1 was detected in enterocytes, goblet cells and Paneth cells in both IODMs and CODMs (Figure 3A). At baseline, OAS1 levels were significantly higher in CODMs compared to IODMs (Figure 3B,C). Additionally, OAS1 expression was elevated in goblet cells relative to enterocytes across both intestinal segments (Figure 3D). Crucially, BB-12 treatment selectively enhanced OAS1 expression within the ileal enterocyte population (Figure 4A–C). In contrast, LGG did not elevate total OAS1 but selectively increased expression in ileal ECs, with a similar trend in colonic ECs (Figure 4A). This targeted induction demonstrates that probiotics do not merely “inflame” the epithelium but rather calibrate the antiviral defenses of specific cell subsets.

### 3.4. Probiotic Pretreatment Restricts Influenza A Replication in IODMs

**Figure 5:**
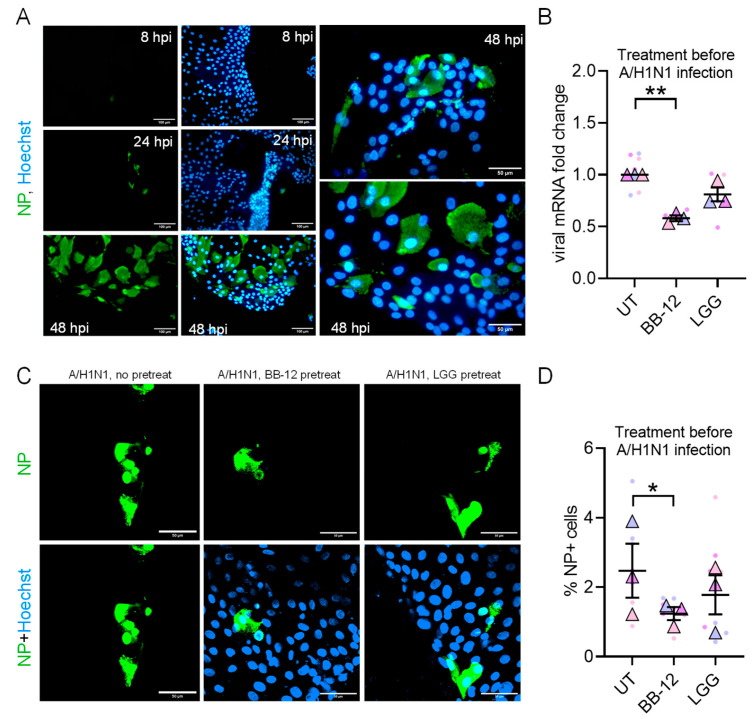
**Restriction of Influenza A/H1N1 replication by BB-12 pretreatment.** (**A**) Representative kinetic analysis of IAV replication in IODMs (MOI 1) at 8, 24, and 48 h post-infection (hpi). Green: viral NP; Blue: Nuclei. Scale bars, 100 μm and 50 μm. (**B**) Quantification of viral replication by RT-qPCR in culture supernatants from untreated and probiotic-pretreated IODMs at 24 hpi. Data are expressed as fold change relative to untreated controls (UT) (2^−ΔΔCt^). (**C**) Representative images showing the reduction in NP-positive cells in probiotic-pretreated monolayers at 24 hpi. Scale bars, 50 μm. (**D**) Percentage of NP-positive cells determined from confocal images. Quantitative results in (**B**,**D**) represent three independent biological donors (*n* = 3) shown as superplots (small circles: technical replicates; large triangles: donor means). Bars represent mean ± SEM. Statistical significance was determined by one-way ANOVA with Tukey’s post hoc test; * *p* < 0.05, ** *p* < 0.01.

To determine if the observed molecular priming translates to functional protection, we infected IODMs with Influenza A/H1N1. IODMs supported productive viral replication, as evidenced by the progressive increase in viral nucleoprotein (NP) expression from 8 to 48 h post-infection (hpi) (Figure 5A). Pretreatment with BB-12 significantly reduced viral replication at 24 hpi (Figure 5B–D). The 24 h time point was selected to capture early differences in viral replication before the onset of extensive influenza-induced cytopathic effects. At later stages of infection (e.g., 48 hpi), progressive epithelial damage can compromise monolayer integrity and complicate quantitative comparisons between conditions. This reduction in viral replication coincides with BB-12-mediated induction of OAS1 and other interferon-stimulated pathways, consistent with the establishment of a probiotic-induced antiviral state.

## 4. Discussion

This study demonstrates that human intestinal ODMs represent a physiologically relevant system to dissect probiotic–host interactions. By using well-characterized probiotic strains, BB-12 and LGG, we show that probiotics induce interferon-associated antiviral responses in a cell type-specific manner. Specifically, we identify OAS1 as a candidate epithelial marker of probiotic-induced antiviral priming and provide evidence of productive influenza A replication in primary human intestinal epithelial cells.

The use of ODMs represents a significant advancement over traditional immortalized cell lines like Caco-2, which are limited by their monoclonal nature, aberrant proliferation, and blunted innate immune responses [21,28,29]. Unlike these transformed models, ODMs preserve the full repertoire of epithelial lineages and maintain an intact interferon (IFN) signaling architecture. Our observation of cell type-specific OAS1 regulation—where goblet cells exhibit higher basal levels and BB-12 selectively enhances expression in enterocytes—highlights biological nuances that are entirely obscured in homogeneous cell lines. Our study advances the field by demonstrating that probiotic-induced antiviral priming is highly cell type-specific. This level of resolution is invisible in traditional cell models and underscores the unique value of primary human models to accurately map the mucosal “antiviral landscape”.

A major finding of this work is the regional and cell type-specific regulation of the antiviral effector OAS1. We observed that BB-12 elicits a stronger antiviral program than LGG, particularly in the ileum. This regional specificity is consistent with reports of a more robust induction of interferon-stimulated genes (ISGs) in the small intestine compared to the colon [30] and suggests that certain probiotics may be uniquely suited to calibrate the “tonic” IFN signaling required for mucosal homeostasis [31,32].

Mechanistically, we propose that epithelial cells recognize microbe-associated molecular patterns (MAMPs) on the probiotic surface through pattern recognition receptors (PRRs), such as Toll-like receptors. This transient interaction may trigger a low-level or “tonic” interferon signaling cascade. The biological function of this process is thought to contribute to maintaining a basal antiviral readiness at mucosal barriers, enabling the epithelium to respond more rapidly to viral invasion. While we identify OAS1 as a robust marker of this induction, it operates within a broader interferon-driven antiviral network rather than acting as an isolated effector. Indeed, as shown in Figure 2, several other critical pathways, including *IFITM* family members, *PKR (EIF2AK2)*, and *STAT1/2* signaling, were concurrently upregulated. Viral restriction may reflect the combined activity of this synergistic antiviral program. However, OAS1 is highlighted in our study because it displayed robust and spatially distinct induction in specific epithelial subsets, making it a particularly sensitive candidate biomarker reflecting the activation of this broader defense program.

The induction of OAS1 is particularly relevant, as this enzyme activates RNase L to degrade viral RNA, and its genetic variability is a known determinant of viral infection outcomes [33,34]. By elevating OAS1 levels prior to viral encounter, BB-12 is associated with an increase in the epithelial “antiviral set-point” of the epithelium, a form of prophylactic priming that parallels the protective effects of IFN pre-activation observed in other organoid models [22,35].

Our study also clarifies the role of the gut as a site for Influenza A replication. While typically considered a respiratory pathogen, clinical evidence of gastrointestinal symptoms and fecal viral shedding in flu patients suggests an underappreciated intestinal tropism [4,6]. We demonstrate that primary ileal enterocytes support productive H1N1 replication, which increases significantly between 24 and 48 h post-infection. Early studies reported replication of Influenza A in human intestinal cell lines such as HT-29 [36], but whether primary human intestinal epithelial cells could support replication remained unclear. This supports a biologically plausible framework to explain gastrointestinal manifestations of influenza, such as diarrhea, which may result from direct epithelial infection or local inflammatory responses [37,38,39]. The ability of BB-12 to restrict viral replication in this model suggests that probiotic-induced intestinal antiviral priming may contribute to broader mucosal protection. However, these observations derive from an epithelial in vitro system and should therefore be interpreted cautiously until validated in controlled human studies.

We acknowledge that the use of organoids derived from IBD patients presents a specific limitation. Although the tissue used for organoid generation was obtained from macroscopically non-inflamed regions, the epithelial background may still differ from that of healthy donors due to underlying disease-related alterations. However, we contend that this model is highly relevant for translational research. The in vitro differentiation process standardizes the environment, effectively removing the acute influence of systemic inflammatory cytokines while preserving the host’s genetic and epigenetic background. At the same time, this system can be viewed as a clinically relevant model of a chronically challenged or “fragile” epithelium. Populations with compromised mucosal barriers are often those most likely to benefit from probiotic-based interventions. Furthermore, the observed Paneth cell metaplasia in colonic monolayers [27] reflects a stable remodeling of the mucosa that allows us to study probiotic efficacy in a clinically realistic setting. Future studies incorporating organoids from healthy donors are necessary to determine if these specific antiviral priming effects are universally conserved across different physiological states.

While our data show a strong association between OAS1 induction and reduced viral replication, we did not directly test the causal requirement of OAS1 using genetic perturbation. Future studies employing targeted approaches such as CRISPR/Cas9 or RNA interference will be required to definitively establish the contribution of the OAS1/RNase L pathway to probiotic-mediated antiviral protection. Additionally, as our model focuses on epithelial-intrinsic immunity, it represents the “first line of defense.” In a physiological setting, this response would be further amplified by the Mucosa-Associated Lymphoid Tissue (MALT) and interactions with the resident microbiota.

These insights have several translational implications. First, probiotic effects are highly strain- and context-dependent [40]. Indeed, our comparative analysis showed that while both benchmark strains induced interferon-associated transcriptional responses, BB-12 consistently elicited a stronger antiviral signature and was associated with measurable restriction of influenza virus replication. This differential response is likely due to strain-specific surface architectures and secreted molecules that differentially engage epithelial PRRs. Differences in cell wall components, lipoteichoic acids, peptidoglycan structures, or metabolite production may dictate the potency of interferon pathway activation. Therefore, antiviral priming appears to be a strain-dependent property rather than a universal feature of all probiotics, supporting the concept of “precision probiotics” tailored to specific host responses. Second, probiotics may serve as prophylactic modulators of mucosal “antiviral set points”. BB-12’s ability to elevate IFN-stimulated genes prior to infection parallels findings where IFN pre-activation protects organoids against adenovirus and astrovirus [22,35]. Finally, BB-12 (BB-12) has an excellent safety record in clinical trials, including pediatric and immunocompromised populations [41,42]. Given this safety profile, BB-12 could potentially be employed as a prophylactic intervention to raise the “antiviral set-point” in vulnerable populations (e.g., the elderly or immunosuppressed), mitigating the severity of viral gastroenteritis or respiratory viruses with intestinal tropism. However, we acknowledge that our findings demonstrate epithelial-intrinsic mechanisms in vitro. Translating these findings into therapeutic applications will require controlled human clinical trials to evaluate biological feasibility, optimal dosage, and in vivo efficacy.

This study has limitations. Single-cell transcriptomic analysis could further refine our understanding of cell type-specific probiotic responses. Moreover, we focused on epithelial-intrinsic immunity; future coculture models with immune and stromal cells are needed to capture mucosal complexity. Finally, while BB-12 reduced Influenza A replication, studies with other enteric viruses (e.g., rotavirus, norovirus, adenovirus) are warranted to define the breadth of protection.

## 5. Conclusions

In conclusion, this work establishes human intestinal ODMs as a physiologically relevant platform to investigate probiotic–host interactions. Our findings indicate that the probiotic strain BB-12 is associated with enhanced epithelial antiviral responses in a cell type-specific manner and is associated with induction of the interferon-stimulated gene OAS1 and restriction of Influenza A replication. These results support the concept that selected probiotic strains can modulate epithelial antiviral readiness and provide a mechanistic framework for future studies exploring probiotics as modulators of mucosal antiviral immunity.

## Figures and Tables

**Table 1:** Primers used for Real Time PCR.

Gene	NCBI Reference Sequence	Forward, 5′-3′	Reverse, 5′-3′	Annealing Temperature (°C)	Concentration Each (nM)
*ACTB*	NM_001101.5	CTGGACTTCGAGCAAGAGATG	AGTTGAAGGTAGTTTCGTGGATG	60	250
*CDX1*	NM_001804.3	GGAGAAGGAGTTTCATTACAG	TGCTGTTTCTTCTTGTTCAC	60	250
*CGAS*	NM_138441.3	TATATACCCTCTGGGAGTGGGA	CCACTGCACTTGGCCTAAAA	60	300
*CHGA*	NM_001275.4	GCTACCCCGAGGAGAAGAAAGA	TCCCGCCCCATACCAACCT	58	250
*FABP1*	XM_054341028.1	ACAGCCTACAGTGGACAGTCT	TCTTCCGGCAGACCGATT	60	200
*IFIH1 (MDA5)*	NM_022168.4	GCATATGCGCTTTCCCAGTG	CTCTCATCAGCTCTGGCTCG	60	300
*IFITM1*	NM_003641.5	TCTGCTGCCTGGGCTTCGTA	AACGATGACGACGATGGCCG	60	300
*IFITM2*	NM_006435.3	GGGTGACTGAGCTGGGGGAA	CTCCAACCACATCGCCCACC	60	300
*IFITM3*	NM_021034.3	CCAGAGAGGTGTCGGTGCCT	GGCACTTAGCAGTGGAGGCG	60	300
*IFNA1*	NM_024013.3	AGAAATACAGCCCTTGTGCCT	ATAGCAGGGGTGAGAGTCTTTGA	60	300
*IFNB1*	NM_002176.4	TCTCCTGTTGTGCTTCTCCACG	CCTCCCATTCAATTGCCAC	60	300
*IRF3*	NM_001571.6	GAGCTGTGCTGGCGAGAAG	CTCTCCAGGAGCCTTGGTTG	60	300
*IRF7*	NM_001572.5	CCATCGGCTTTTGGGTCTGT	TTCCCATGGTCCGGCCTC	60	300
*ISG15*	NM_005101.4	GGCCAGGTTCTAAGTGTGCT	GCGTCACACAGGTTCAGAGA	60	300
*LGR5*	XM_054373693.1	GATGTTGCTCAGGGTGGACT	GGGAGCAGCTGACTGATGTT	60	250
*MAVS*	NM_020746.5	GCAATGCCGTTTGCTGAAGA	CGCCGCTGAAGGGTATTGAA	60	300
*MUC17*	NM_001040105.2	CAATGGAACTGACTGTGAC	CCCGGAATACACAATATTCATC	60	250
*MX1*	NM_002462.5	GGCTGTTTACCAGACTCCGACA	CACAAAGCCTGGCAGCTCTCTA	60	300
*OAS1*	NM_016816.4	TGTCCAAGGTGGTAAAGGGTG	CCGGCGATTTAACTGATCCTG	60	300
*PKR (EIF2AK2)*	NM_001135651.3	TCAGTTTGCCTTCCTGGATTTGT	TTCTTCCCGTATCCTGGTTGG	60	300
*POU2F3*	NG_030035.1	TCTGGTGGATGAGGGGACAA	GCCTCGAGCCTTTGGTTCTT	60	250
*REG3A*	NM_138938.3	GGTGAGGAGCATTAGTAACAGC	CCAGGGTTTAAGATGGTGGAGG	60	250
*STAT1*	NM_007315.4	TGTATGCCATCCTCGAGAGC	AGACATCCTGCCACCTTGTG	60	300
*STAT2*	NM_005419.4	CCGGGACATTCAGCCCTTTT	CTCATGTTGCTGGCTCTCCA	60	300
*TLR3*	NM_003265.3	TGCCGTCTATTTGCCACACACTTCC	ACAGCATCCCAAAGGGCAAAAGG	62	75
*TLR7*	NM_016562.4	TGCTCTGCTCTCTTCAACCAGACC	ACCATCTAGCCCCAAGGAGTTTGG	64	250
*TLR9*	NM_017442.4	GGACCTCTGGTACTGCTTCCA	AAGCTCGTTGTACACCCAGTCT	60	100

**Table 2:** Antibodies used in the study.

Antibodies	Catalog Number	Host	Clonality	Working Dilution	Supplier
**Primary Antibodies**					
anti-SI	ab224085	rabbit	poly	1:200	Abcam (Cambridge, UK)
anti-Lysozime	pa5-16668	rabbit	poly	1:500	Invitrogen (Carlsbad, CA, USA)
anti-Chromogranin-A	sc-393941	mouse	mono	1:100	Santa Cruz Biotechnology (Dallas, TX, USA)
anti-Mucin 2-Alexa Fluor 488	sc-515032	rabbit	mono	1:100	Santa Cruz Biotechnology (Dallas, TX, USA)
anti-OAS1-Alexa Fluor 594	sc-374656	mouse	mono	1:100	Santa Cruz Biotechnology (Dallas, TX, USA)
**Secondary Antibodies**					
Anti-Mouse IgG (H+L)-Alexa Fluor 488	115-545-003	goat	poly	1:100	Jackson ImmunoResearch Laboratories (West Grove, PA, USA)
Anti-Mouse IgG (H+L)-FITC	AP124F	goat	poly	1:100	Chemicon International (Temecula, CA, USA)
Anti-Mouse IgG (H+L)-Cy3	115-165-062	goat	poly	1:100	Jackson ImmunoResearch Laboratories (West Grove, PA, USA)
Anti-Rabbit IgG (H+L)-Alexa Fluor 647	A-21441	rabbit	poly	1:500	Invitrogen (Carlsbad, CA, USA)
Anti-Rabbit IgG (H+L)-Alexa Fluor 488	A-11008	goat	poly	1:100	Invitrogen (Carlsbad, CA, USA)
Anti-Rabbit IgG (H+L)-Alexa Fluor 594	A-21442	chicken	poly	1:100	Invitrogen (Carlsbad, CA, USA)

## Data Availability

The original contributions presented in this study are included in the article/Supplementary Material. Further inquiries can be directed to the corresponding authors.

## Supplementary Materials

The following supporting information can be downloaded at: https://www.mdpi.com/article/10.3390/microorganisms14040751/s1, Table S1: Donors’ characteristics.

## Abbreviations

The following abbreviations are used in this manuscript:

BB-12	*Bifidobacterium animalis* ssp. *lactis* BB-12
CODMs	Colonic ODMs
IAV	Influenza A Virus
IBD	Inflammatory Bowel Disease
IODMs	Ileal IODMs
LGG	*Lacticaseibacillus rhamnosus* GG
ODMs	Organoid-derived monolayers
