# Supplementary material for: Bifidobacterium animalis subsp. lactis BB-12 Primes Epithelial Antiviral Defenses and Restricts Influenza A Virus Replication in Human Intestinal Organoid-Derived Monolayers
Source: Microorganisms. 2026 Mar 27;14(4):751. doi: 10.3390/microorganisms14040751 (PMC13118500; doi:10.3390/microorganisms14040751)
Supplement: Supplementary file 1 [file microorganisms-14-00751-s001.zip › microorganisms-4175410-supplementary.pdf]

Supplementary Table S1. Donors' characteristics.

Donor number	Age	Sex	Disease	Severity	Tissue	
					**Ileum**	**Colon**
**1**	37	female	UC	Mayo score 2	x	--
**2**	29	male	CD	Harvey-Bradshaw score 7	x	--
**3**	66	male	CD	Harvey-Bradshaw score 8	x	--
**4**	20	female	CD	Severe	--	x
**5**	46	male	CD	not available	--	x
**6**	66	female	UC	Mayo score 3	--	x
